# A Model of Perception of Privacy, Trust, and Self-Disclosure on Online Social Networks

**DOI:** 10.3390/e21080772

**Published:** 2019-08-07

**Authors:** Lili Nemec Zlatolas, Tatjana Welzer, Marko Hölbl, Marjan Heričko, Aida Kamišalić

**Affiliations:** Faculty of Electrical Engineering and Computer Science, University of Maribor, Koroška 46, 2000 Maribor, Slovenia

**Keywords:** online social networks, Facebook, privacy, self-disclosure, trust, model

## Abstract

Online Social Networks are used widely, raising new issues in terms of privacy, trust, and self-disclosure. For a better understanding of these issues for Facebook users, a model was built that includes privacy value, privacy risk, trust, privacy control, privacy concerns, and self-disclosure. A total of 602 respondents participated in an online survey, and structural equation modeling was used to evaluate the model. The findings indicate significant relationships between the constructs in this study. The model from our study contributes new knowledge to privacy issues, trust and self-disclosure on Online Social Networks for other researchers or developers of online social networks.

## 1. Introduction

Online Social Networks (OSNs) are an important communication form, and in recent years have been a topic of interest for researchers. OSNs have been defined as a networked communication platform where users (1) host their profiles, including user-supplied content, other user-supplied content and system-supplied data; (2) connect with users; and (3) view or interact with user-generated content provided by user connections on OSN [1]. Facebook, the most popular OSN, has all the features included in the OSN definition. Users have their profiles set to different accessibility levels, they are in contact with friends on Facebook, and they can see the activities of users of Facebook, depending on the accessibility levels of the user’s profile. Facebook was established in 2004, and in 2009 it became the most popular OSN [2,3]. Facebook reported 2.38 billion active monthly users in March 2019 [4].

This study addresses Facebook privacy issues, trust, and self-disclosure. Privacy is a personal boundary regulation process that regulates private information, according to context [5,6]. Trust encompasses how trustworthy the user feels that an OSN is. The act of revealing personal data to others was defined as self-disclosure [7].

The purpose of the study was to examine whether there are any links between privacy issues, trust and information disclosure on OSNs, especially Facebook. Studies researching issues of privacy, trust and disclosure of personal information on Facebook have already been published (see Section 2), but to the best of our knowledge no studies have yet examined the constructs presented in this study. This study seeks to link these issues in research to a more complete view of privacy value, privacy risk, privacy control, privacy concerns, trust in Facebook, and self-disclosure on OSNs through development of a model of relationships between the set of constructs. The model results were gained from responses to an online questionnaire, where users self-reported on their Facebook use, privacy concerns, trust, and self-disclosure.

The relationships between the constructs in the research model will be examined by using Structural Equation Modeling (SEM). First, the model analysis was done, and the SEM results are presented after all the steps of model analysis. SEM is designed to evaluate model fit and research hypotheses [8]. The generalized maximum entropy was already used a few times for the improvement and analysis of SEM models when the sample was not big enough [9,10,11]. The sample in our study includes Slovenian Facebook users aged 18 to 63, and 939 users completed an online survey (administered with the use of the LimeSurvey tool), from which 602 final samples were used in the final analysis of our model.

The contribution of this study is to create a link between the variables privacy issues and trust, and examine their impact on OSNs’ self-disclosure, which have not been considered together before. In addition, our study will provide OSN providers and users with a better understanding of how OSN users can share more information. 

The rest of the paper is organized as follows. Section 2 discusses existing literature on privacy and self-disclosure issues. Section 3 defines and hypothesizes our research model; also discussed are the constructs used in our model. Section 4 describes the research methods, where the data collection is explained, while also looking at the measures for our model. Section 5 explains the data analysis and presents the results. Section 6 concludes the paper, including a discussion of the findings and their practical implications.

## 2. Literature Review and Theoretical Development

### 2.1. Previous Work on Privacy, Trust, and Self-Disclosure of Osns

In previous studies, privacy, trust, and self-disclosure on OSNs among users has been a topic of research. Privacy is the most widely investigated research topic within these three areas of OSNs. Privacy means that individuals choose what information they share and with whom, using the privacy controls on OSNs, which has been explained similarly for offline conversations in Communication Privacy Management theory [12,13]. The Communication Privacy Management theory was also used in the investigation on privacy issues associated with Facebook apps, where researchers found that the collection of information, the monitoring of profiles and general privacy concerns affect users’ privacy concerns and, with this, also their willingness to share their profiles with a Facebook app, but, on the other hand, that users also often misunderstand what data Facebook apps are collecting on them and their settings do not correspond to their privacy concerns [14,15]. In regard to privacy control, users usually have more closed profiles on Facebook if they spend more time on Facebook, and if their friends have closed profiles [16]. The friend reference is also important when we take into account the Social Identity model of Deindividuation (SIDE), according to which, an individuation condition in a group will be lower when the polarization towards group norm is measured than when a group is deindividuated [17]. In one study they have also found that the recency of contact between friends predicts the strength of a tie between friends [18]. On the other hand, research has shown that people disclose information almost independently of their concerns about privacy [19,20,21]. Perceived privacy risk explains how the user interprets the risk of posting their personal information on OSNs. Privacy risk affects the intention of disclosing a user’s location, and its intention to disclose via a mobile application in two studies [22,23]. Privacy value is also an important part of privacy, where the user expresses how they feel their personal information should be kept on OSNs. Privacy value was considered to have a significant impact on the users’ privacy concerns and their behavioral intention of further website use [24]. Also, computer anxiety, self-esteem, and consumer alienation had a high impact on consumers’ concern for information privacy [25].

Trust is another factor that was highly researched with regard to OSNs [26]. Trust in Facebook has been shown to affect users’ privacy concerns directly [27]. In another study, trust was highly influenced by security and privacy, and had a strong impact on the users’ attitudes and intention to use OSN [28]. Trust also affects users’ behavioral intention for mobile payment services [29].

Privacy constructs were also linked to the self-disclosure behaviors of users of OSNs. In previous studies, the direct impact of privacy concerns on Facebook self-disclosure behaviors was confirmed [30,31]. Privacy concerns and awareness also affect the intention of users’ self-disclosure on OSNs [32,33]. Another study showed the effect of trust in service providers, and privacy risk on the self-disclosure of users of microblogging websites [34]. Research has also shown that frequent OSN use and parents’ educational influence motivate users to increase their concerns about privacy and disclose less information on Facebook [35]. Using the SIDE model, the researchers in one study also found that self-disclosure in shy people can be increased by deindividuation [17,36]. In another paper, many factors have been identified that have an impact on information disclosure on Facebook: Number of friends, benefits, time spent on Facebook, personality factors, perceived risks, social cohesion, and the need for popularity, and meeting new people [37,38,39].

We have carried out an examination of previous studies in this section and the studies are showing the link between certain constructs within the models. The links will be examined more thoroughly further on in the privacy, trust, and self-disclosure on OSNs and websites models section of this paper. But first, the theory that some of the models from previous studies described in this section were based on is the Communication Privacy Management theory, and it is described in the next section in more detail. 

### 2.2. Petronio’s Communications Privacy Management Theory

The theory we used as the basis for our research model formation is Petronio’s Communication Privacy Management (CPM) theory [13]. The process of opening up and closing borders to others is defined as privacy in CPM. In the first step, the information ownership is essential, because, when a person shares their private information, the co-ownership rights for private information are broadened to other users. The ownership could only be transferred if the information is shared with the permission of the owner, not if it is collected without permission. Secondly, the CPM theory states that control of privacy is important for individuals, which means they want the option of disclosing or concealing private information when they disclose information. Thirdly, boundary turbulence is considered to occur when, without the permission of the owner, some information that should not be shared has been shared. This might be caused by the disruption of privacy management and relational trust. By binding the CPM theory to OSNs, we can resume that, when they publish the information, Facebook users can control who has access to the information they publish and whom they trust. Furthermore, as predicted by the CPM theory, their friends can also further share their information, and, in ideal cases, trust between Facebook friends should not be breached. 

In previous OSN privacy, trust and disclosure research we can also find the CPM theory [40,41,42,43,44]. The following section presents models for privacy, trust, and self-disclosure on OSNs and other websites, some of them based on CPM theory.

### 2.3. Privacy, Trust, and Self-Disclosure on Osns and Websites Models

For building our model, we selected the most suitable privacy, trust and self-disclosure constructs from the literature review to explain the link between the constructs in our model. Table 1 presents a summary of constructs, user groups and references for all papers that were analyzed thoroughly before our research model was developed.

In two similar studies by the same authors, two models of user privacy concerns were proposed-both studies examined a complex set of privacy issues and tested website users of e-commerce, social networking, finance, and healthcare [45,46]. The models are based on the theory of CPM and are tested with the analysis of Partial Least Squares (PLS). Empirically, the models support understanding the formation of privacy concerns of an individual. In the first model [45] with the users of OSNs, 49% of the variance is explained in privacy concerns. The direct effects on privacy concerns are the privacy risk, perception of intrusion, and privacy control, while the disposition to privacy has a direct effect on all three above- mentioned constructs. The perceived effectiveness of privacy policy also has a significant effect on privacy control and risk. The privacy seal does not affect the risk of privacy significantly, but has a significantly positive effect on privacy control. Further on, privacy awareness has a nonsignificant effect, whereas privacy social norms have a significantly positive effect on the disposition for privacy. In the second model [46] with the users of OSNs, 40% of the variance is explained in privacy concerns. This is similar to the first model explained, with the constructs perception of privacy, privacy awareness, and privacy social norm omitted from this model. All the effects in this model were also confirmed in the first model.

Another study confirmed that the vulnerability of resources privacy risk, the severity of the threat, privacy intrusion, have a positive effect on those non-using privacy controls, while the cost of not using privacy controls had a positive effect on the attitudes of Facebook users [47]. Further on, social norms, perceived behavioral, and attitude controls perceived as mediator variables had a positive impact on the user’s intention of privacy control use. The variance explained in this study was 36% for attitude, 31% for the cost of not using privacy controls, and 24% for intention to use privacy controls. Their model implies that the attitude of individuals towards using Facebook’s privacy controls is influenced by the use of privacy controls and the cost of using privacy controls. 

Another study was done using PLS analysis among website users, where 70% of the variance was explained for the behavioral intention [48]. A direct effect of perceived benefits and site-specific privacy concerns was confirmed on behavioral intention. Privacy experience had an effect on disposition to privacy, and, together with two other constructs (website reputation and website familiarity), these constructs had an effect on the site-specific privacy concerns construct, also explaining 21% of the variance for this construct.

McKnight, et al. [49] tested a privacy calculus model for Facebook users, and confirmed an effect of privacy concerns, trusting beliefs and information sensitivity on information disclosure. 23% of variance was explained for information disclosure. Variables perceived usefulness and enjoyment had a direct effect on continuance intention, which explained 42% of the variance for this construct. 

Chen [50] analyzed OSN users’ privacy and self-disclosure behaviors. The theoretical model identified the impact on behaviors related to the privacy of personal information by perceived criticism, extroversion and perceived internet risk. His model also shows that the value of privacy reduces the impact of attitudes on behaviors of self-disclosure. The SEM model tested on OSN users which indicated the relationships between the proposed constructs, was also presented in a study on self-disclosure [51]. Their model implied that users are less concerned about their privacy, and that the perceived enjoyment has a significantly positive impact, and that privacy concerns have a considerably negative effect on OSN’s self-disclosure.

In another study, the effect of the online privacy policy was also investigated on the willingness to provide personal information on websites [52]. The researchers divided the privacy policy construct into access, notice, security, choice, and enforcement. The impact was examined on online privacy concerns and trust of all individual connections. The paper shows the links between the constructs, also indicating the negative effect of privacy concerns on willingness to provide personal information.

The research model presented in this article is based on the models from Table 1. Privacy value, privacy risk, trust in Facebook, privacy controls, privacy concerns, and self-disclosure constructs were selected for the purpose of developing this research model. These constructs appeared in the analyzed models often, and presented a base for building our model. Previously developed and described models were not tested only on OSNs users, but also on websites and general Internet users. The aim of the paper was to create a model for Facebook and OSNs users, in order to analyze the interaction between privacy, trust, and self-disclosure on OSN.

## 3. Research Model and Hypotheses

**Figure 1:**
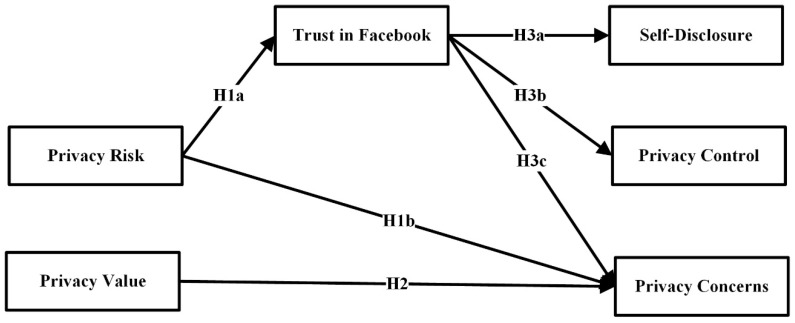
The Online Social Networks research model of privacy issues, trust, and self-disclosure.

Figure 1 presents the research model of this study, and was developed based on the basis of previous research on privacy issues, trust, and self-disclosure. Privacy value and privacy risk are independent constructs in our model. Privacy value measures how users feel about privacy, and how important it is to preserve their privacy. Privacy risk is a construct that measures how the user feels their information is being used by Facebook, and how much personal information they provide to Facebook.

Constructs influenced by the mentioned constructs are trust in Facebook, privacy concerns, privacy control and self-disclosure. Trust in Facebook measures how confident the user feels Facebook is and what kind of reputation it has. Privacy control measures the extent to which users believe they have control over who can access their profiles information. Privacy concerns measure if users are concerned about other users who will have access to the information they post on OSNs. Self-disclosure measures the extent to which the profiles of users are filled with their personal information and how much information users disclose on Facebook.

Our model in Figure 1 proposes that privacy risk affects trust in Facebook and privacy concerns. Privacy value has an impact on privacy concerns. We also hypothesize that trust in Facebook affects self-disclosure on Facebook, privacy control and privacy concerns. Further on, all the constructs in our research model are discussed in more detail, followed by the hypotheses.

### 3.1. Privacy Risk

Privacy risk is a widely used construct in other studies, and explains to users the risk to their privacy while putting personal information on OSNs [22,37,45,46,47,50,53,54,55]. In a study by Dinev and Hart [54] on users of e-commerce transactions, an effect of privacy risk on Internet trust was confirmed. Three studies among OSN users have also confirmed the impact of privacy risk on concerns about privacy [31,45,46]. Therefore, the following hypotheses were proposed concerning the construct of privacy risks:

**H1a.**  *Privacy risk has a negative impact on trust in Facebook.*

**H1b.**  *Privacy risk has a positive impact on privacy concerns.*

### 3.2. Privacy Value

In OSNs, the privacy value construct is an important issue, also referred to as disposition to privacy, and explains how users feel about privacy threats, privacy in general, and how important it is to maintain their privacy [24,28,45,46,48,50,56]. Research among students by Acquisti and Gross [20] showed that Facebook members are less concerned about personal privacy risks than non-Facebook members. That implies that non-members had already become more aware of privacy threats in the early days of the Facebook site, which resulted in a greater value for their privacy. Research has already confirmed the effect of privacy concerns and privacy value between web users [24,48] and OSN users [45,46]. Another study on Facebook information disclosure revealed that some users are not concerned about posting their personal data, but most users worry about their identity and how they value their privacy, which also influences how they use Facebook [57]. A study of students who use Facebook showed that due to concerns about who could access this information, they do not want to see their information revealed on Facebook [58].

The literature suggests that privacy value has an impact on privacy concerns. Therefore, this study proposes the following hypothesis:

**H2.**  *Privacy value has a positive impact on privacy concerns.*

### 3.3. Trust in Facebook

Another construct that was analyzed was trust in websites, in particular on the OSN Facebook. Trust in websites is a construct used in many other studies [24,28,30,37,49,52,54,59,60,61]. This construct explains if users trust the website, in our case Facebook, and if they will recommend it to others. Trust was found to have a significant impact on self-disclosure of users. In some studies, the impact of trust on self-disclosure was positive, meaning that when the users have higher trust in a website, more information will be disclosed on that website [30,37,52,54,62]. On the other hand, one study has found a negative impact of trust on self-disclosure, meaning that the higher the trust of a user for the website, the less they will disclose [49]. An important factor to consider regarding self-disclosure and trust is also the results of some surveys showing that, when a person is anonymous and his identity is salient (e.g., a person is interacting within a specific group on OSN), the person’s trust in the platform is increased, as well as self-disclosure [17]. By using entropy for assessment of information availability, the authors of the studies have found that by using computer-mediated communication, the normative influence becomes ineffective when the individuals are deindividuated [63,64]. Based on the results of the previous studies, trust in Facebook should have an effect on self-disclosure. Since the studies show contradictory results of positive or negative impact, we will test if the deindividuated theory stands in this case, and we will test if the path in the model is negative. 

In a study by Li [48], the author found a significantly negative impact from website reputation and website familiarity on privacy concerns. This significantly negative effect of trust on privacy concerns was also confirmed in three other models of OSN users [30,61,65]. As explained in these studies, trust in a website has an effect on the privacy concerns of Facebook users. 

We also hypothesize that trust in Facebook has an effect on privacy control, which was not researched in previous studies. The theory of CPM implies that it should be possible to manage private information. This should also increase trust in a website. An example of such manageable information is when the relationship between an individual and a friend on Facebook changes and changes the privacy settings of their disclosed information to this friend or even unfriend someone [66]. Studies have also shown that individual and group privacy protection rules are needed in order to gain better control of data [13,44]. In one study they have found that some users create new accounts on Facebook to see how others see their Facebook account [67]. The following hypotheses are proposed:

**H3a.**  *Trust in Facebook has a negative impact on self-disclosure.*

**H3b.**  *Trust in Facebook has a positive impact on privacy control.*

**H3c.**  *Trust in Facebook has a negative impact on privacy concerns.*

The research methods used in this survey, data analysis, and the results of this study are described in the following chapters.

## 4. Research Methods

To test our six hypotheses, our study used an online questionnaire. The survey questions were developed based on existing literature and following discussion with other Faculty members for each construct–privacy value, risk to privacy, trust on Facebook, control of personal data, privacy, and self-information. 

### 4.1. Data Collection and Participants

Facebook users in Slovenia have been targeted by this research. Slovenia’s Facebook penetration in December 2017 was 44% [68]. Our study participants were between the ages of 18 and 63. Using a convenience sampling, participants were recruited and a call for participation was posted on various Facebook groups, web forums, and sent to students at the home institution of the researchers via e-mail. The questionnaire’s welcome page notified the participants of the research title, data protection and treatment. The survey had a total of 44 questions.

There were 939 participants who entered the survey, out of which 727 completed the survey. One-hundred-and-fourteen cases have been excluded after a case screening because they have not used Facebook and 613 participants have been analyzed further for uncommitted responses or errors. The case was excluded if the Standard Deviation for each person’s responses was less than 0.5, since there was no variation in these cases. We found 8 such unengaged respondents who gave the same answer to all Likert scale questions and deleted those responses. The age and educational variables have been tested for outliers, and no cases have been excluded, since no outliers were found in boxplots. With the analysis of Structural Equations Modeling, another 3 cases were removed due to abnormal Cook’s distance [69]. Six-hundred-and-two cases were valid and used for analysis after complete screening. A detailed demographic sample for valid cases is shown in Table 2.

### 4.2. Measures

In order to ensure validity measurement, items were combined from existing measures for the constructs. Each construct was evaluated with items that ranged from (1) strongly disagree to (7) strongly agree on a Likert 7-point scale. Table 3 provides detailed structures and references for the construction of constructs. The survey contained 24 items. With 15 Facebook users, the survey instruments were pretested and refined and validated for statistical properties. 

## 5. Data Analysis and Results

A data analysis was conducted using IBM SPSS Statistics 23.0 and AMOS 23.0 software. Structural Equations Modeling was used and testing of hypothesis was performed.

### 5.1. Model Analysis

First, variable screening was performed for missing data, and then a factor loadings analysis was performed and iterated until a clean pattern matrix was reached. Five variables were dropped because of a loading lower than 0.5. These were SD5 (Variable 5 for the Self-disclosure construct), PCt3 and PCt4 (Variables 3 and 4 for the Privacy control construct), PV1 (Variable 1 for the Privacy value construct), and TR4 (Variable 4 for the Trust construct). A total of 19 items remained in our model.

With Cronbach’s alpha, a commonly used measure testing the extent to which multiple items for a construct belong together we evaluated convergent validity. The coefficient varies between 0 and 1. The Cronbach’s alpha in our research model ranged from 0.772 to 0.901. The acceptable coefficient of reliability is above 0.7, although some authors claim that the coefficient can be above 0.6 when doing exploratory analysis [72,73]. For each construct, Cronbach’s alpha was calculated, taking into account all the items left after five items were excluded. In Table 4, a summary is presented of all values.

Table 4 also shows the factor loadings for the final set of items in our model. The results show that the research instrument is highly internally consistent and has high factor loadings, and is therefore reliable.

A confirmatory analysis of our model factor was then carried out. Table 5 shows the results of the model fit for the initial measurement model. We have included the following four fit indices; the Goodness-of-Fit (GFI), the Comparative Fit Index (CFI), the Normed Fit Index (NFI), and the Root Mean Square Error of Approximation (RMSEA). The recommended values in the Table were adapted from [74], Chin and Todd [75], and Hair [76]. All the values are within the recommended value.

The Composite Reliability (CR), Average Variance Extracted (AVE), and factor correlations matrix are shown in Table 6 for the validity and reliability of our model. The Composite Reliability values are all surpassing the minimum value of 0.7, being between 0.864 and 0.944, [76]. The extracted Average Variance values ranges from 0.587 to 0.689, with 0.5 being the minimum value [76], all exceeding the recommended minimum value. The AVE value exceeds the square correlation between different constructs, and the discriminating validity criteria are confirmed [77]. There is good discriminating validity for all constructs, as in Table 6. To sum up, our model does not have any concerns about reliability or validity.

For the structural analysis, we first did multivariate assumptions for outliers and an influential using Cook’s distance analysis [69]. Three cases exhibited abnormal Cook’s distances and we opted to remove them in the phase of structural analysis. Next, a multicollinearity test was done, using the Variance Inflation Factor (VIF), which should range between values 1 and 4, and all the results were within these values [78].

Next, hypotheses were tested using the paths.

### 5.2. Testing Research Hypotheses

**Figure 2:**
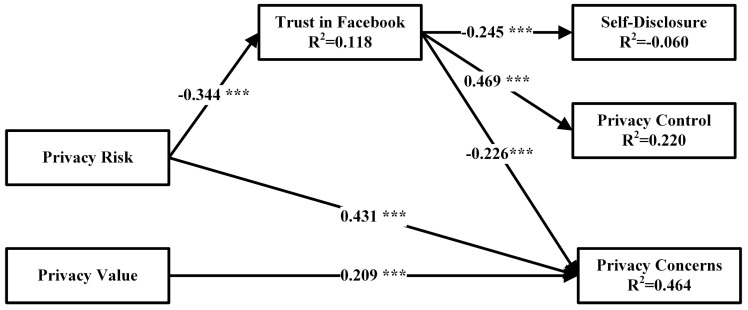
The path coefficient analysis.

Our model was tested for the overall fit of the model, as well as the model also tested individual paths. Figure 2 shows the results of the path analysis for the links between various groups of factors.

The R-squared value in the model presents the amount to which the dependent variable is explained by the independent variables. In our model, the privacy concern is explained to 46.4% by privacy risk, privacy value, and trust in Facebook. This shows that almost half of the construct privacy concern is explained by the model presented in this paper. Trust in Facebook explains privacy control by 22% and self-disclosure by 6%. Trust in Facebook is explained by privacy risk for 11.8%. In our previous study privacy concerns were explained for 38.1%, but a bigger focus was set to self-disclosure, where 32.6% of variance was explained [33]. To put this result into perspective, we have focused more on the privacy concerns construct in this model, and it was also explained very highly by the independent variables in the model.

The analysis of the path coefficient and the results of the t-statistics explain the developed hypotheses. The significance and strength of each path is assessed by the standardized coefficient (β) and by a *t* value, which needs to be above 2.0 or below −2.0 [59]. Table 7 shows the results of the path analysis and hypotheses testing. The results indicate that all the paths in our model are significant at *p* lower than 0.001, and all *t* values above 2.0 and below −2.0.

Privacy risk has a significant impact on trust in Facebook, and privacy concerns with standardized value −0.413 and 0.627 and high *t* values lower than −6.0 or higher than 7.0 respectively. Privacy value has a significant impact with β at 0.231, and *t* values above 4.0 on privacy concerns. Trust has a significant impact on self-disclosure, privacy control and privacy concerns and standardized values −0.284, 0.355, and −0.274 respectively, with *t* values lower than −5.0 and higher than 7.0. All the impacts are significant, and will be discussed in the next section.

## 6. Discussion

Facebook and other OSNs are part of many people’s everyday lives. OSNs users publish a lot of personal information on OSNs daily. The spread of OSNs has opened up new issues in the perception of users on OSNs about privacy, trust and self-disclosure.

The goal of this study was to develop a model of how the value of privacy and perceived privacy risk affects the trust of OSNs, privacy control, privacy concerns, and self-disclosure on OSNs by the users. The model in this study was built based on previous research done in the field of Privacy, Trust and Disclosure. Online data collection methods were used in order to validate our model. The survey was attended by 602 respondents between the ages of 18 and 63, who use Facebook and are from Slovenia.

Our results provided the model with the six constructs: privacy value, privacy risk, and trust in Facebook, privacy control, privacy concerns, and self-disclosure. These were tested on users of Facebook. First, the initial measurement model was built, which was then analyzed and adjusted to the final measurement model. SEM analysis has confirmed the final research model, and five out of six test hypotheses have been verified by the results of the path analysis. Privacy value and privacy risk are independent constructs in our model, and both have positive impact on privacy concerns, while privacy risk also has a negative effect on trust in Facebook. The mediator construct trust in Facebook has a direct impact on self-disclosure, privacy concerns, and privacy control constructs. 

In order to conceptualize our results, the privacy risk was shown to have a negative impact on Facebook trust and a positive impact on privacy concerns. Privacy risk was shown to have a negative effect on trust in a website, which is in line with previous studies [54,79]. The positive effect of privacy risk on privacy concerns was also confirmed in other studies [31,45,46]. These hypotheses propose that the more the user believes it is risky to give their information on Facebook, the lower trust in Facebook the user will have, and the higher their privacy concerns for their personal information will be.

In other studies, privacy value was found to have a positive impact on privacy concerns [24,45,46,48]. This impact describes that the more the user values their privacy, the higher their concerns for privacy will be.

Trust in Facebook was found to have a negative effect on Facebook self-disclosure. We have hypothesized that trust in Facebook would have a negative impact on self-disclosure, but some studies have shown a positive impact. The negative path was also found in one previous study [49], whereas, in some studies, it was confirmed as having a positive impact [37,52]. As debated already in the hypotheses construction, the SIDE model assumptions might explain the negative impact, because when a person’s social identity is salient, both trust and self-disclosure increase [17]. It has also been found that trust in Facebook has a significantly positive effect on privacy control and a negative impact on privacy concerns. Other studies have also found a similarly significantly negative impact for trust in Facebook on privacy concerns [48,61], and we did not find any other study that would test the effect between trust in a website and privacy control. These three hypotheses propose that the more trust the user has in Facebook, the more information they will disclose, and the less privacy concerns they will have. Also, the user will feel that they have higher privacy control if the trust in Facebook is higher.

The results of our research in the field of privacy, trust and self-disclosure on OSNs correspond with previous results from research in the majority of instances, and form a new model with good overall model fits. The results added new meaning to research in the field of the formation of trust in Facebook, privacy controls and concerns, as well as self-disclosure on OSNs. Although some of its paths have already been confirmed in previous studies, such a model was not yet built, so this model presents new knowledge in understanding the users’ perception of privacy, trust, and self-disclosure on Facebook. The model can bring better understanding of correlations between different constructs to researchers, and could also be applied to other platforms, not only on OSNs. The model can also help OSNs developers understand how their users feel about their privacy and when they disclose the most information, which is probably the goal of OSNs. To get the most out of this model, we have confirmed the negative impact of trust on self-disclosure by taking into account the SIDE model as a fundamental theory, and we have also confirmed a highly significant path between trust and privacy control which was not studied before.

### Limitations and Future Directions for Research

There are certain limitations to this study. Facebook penetration in Slovenia is 44% [68] or around 910,000 people. The study sample size was 602 Slovenian Facebook users, which is statistically valid, with a confidence level of 95% and margin of error of 3.99%. Our study cannot be generalized to Slovenian Facebook users or to all Facebook users, because of the convenience sampling method. Future research could aim for a larger and more representative sample to be collected.

The constructs in our model chosen by a comprehensive analysis of existing studies are the next limitation, as potential constructs may have remained unselected. Future studies on Online Social Networks should identify and incorporate potentially significant constructs for issues of privacy, trust and self-disclosure.

The collaborating users in our study have self-reported their opinions on questions which could lead to self-reporting bias, and should be avoided in future studies.

Future studies may need to develop an OSN-specific measurement scale. In this study, the constructs were adapted from models with Internet users, electronic commerce, various websites, users of Facebook and OSN.

## 7. Conclusions

To conclude, this research explains the perception of users of OSNs of privacy, trust, and self-disclosure. The model shown in our research was based on previously developed models, but was extended to include the missing linkages between the structures, and was discussed in greater detail. In this example, a holistic view of Facebook privacy and other structures has been presented, which have significant impacts on Facebook’s user trust and self-disclosure.

The study contributes to a better understanding of the dynamics of users’ privacy, trust and self-disclosure on Facebook, since previous studies have not incorporated some of the important constructs in their models. This study combined privacy value, privacy risk, trust in Facebook, privacy control, privacy concerns, and self-disclosure into one model. This study could help OSN providers to obtain more information from their users on their profiles. This study could provide a better understanding of privacy and trust issues and self-disclosure on Facebook. The developed model can also be used for development of other models, and could also be extended and tested on other platforms like mobile applications, not only OSNs.

## Figures and Tables

**Table 1:** Presentation of models on privacy, trust, and self-disclosure

Independent Variables	Mediator Variables	Dependent Variables	Tested on Users Of	Reference
Privacy awareness, Privacy social norm, Perceived effectiveness of privacy policy, Perceived effectiveness of privacy seal	**Privacy risk**, **Disposition to privacy**^a^, **Privacy control**, Perception of intrusion	**Privacy concerns**	Financial and healthcare websites, electronic commerce, and OSNs	[45,46]
Resource vulnerability, Threat severity, **Privacy risk**, Privacy intrusion	Cost of not using privacy controls, Social norm, Attitude, Perceived behavioral control	**Intention to use privacy controls**	Facebook	[47]
Privacy experience, **Website reputation**, Website familiarity, Perceived benefits	**Disposition to privacy ^a^, Site-specific privacy concerns**	Behavioral intention	websites	[48]
**Privacy concern**, Information sensitivity, **Trusting beliefs**, Perceived usefulness, Enjoyment	**Information disclosure ^b^**	Continuance intention	OSNs	[49]
Extroversion, Perceived critical mass, **Perceived internet risk, Privacy value**	Attitude	**Privacy self-disclosure behaviors**	OSNs	[50]
Perceived likelihood, Perceived damage, Privacy enjoyment	**Privacy concerns**	**Self-disclosure**	OSNs	[51]
Privacy policy (Notice, Choice, Access, Security, Enforcement)	**Online privacy concern, Trust**	**Willingness to provide information ^b^**	Internet	[52]

^a^ Named Privacy value in the proposed model from this paper. ^b^ Named Self-disclosure in the proposed model from this paper.

**Table 2:** Sample of demographics (n = 602).

Variable	Analysis Results
Gender	Male 44.0% Female 56.0%
Finished education	Less than high school 1.5% High school 50.7% Bachelor’s degree 24.4% Master’s degree or higher 23.4%
Age	M 26.73 SD 8.21
Number of friends on Facebook	M 441.06 SD 393.54
Percentage of Facebook Friends whom the user doesn’t know in person	M 11.42% SD 19.26%
Average Facebook use per day	0–10 min per day 13.0% 10–30 min per day 35.4% 30–60 min per day 28.0% More than 60 min per day 23.6%
Experience with Internet use (7-point Likert scale)	M 6.35 SD 0.99
Experience with Facebook use (7-point Likert scale)	M 5.88 SD 1.25

**Table 3:** Measurement of variables.

Constructs	Code	Items	References
Privacy risk	PR1	I think giving personal information on Facebook would be risky.	[45,47]
	PR2	Providing my personal information to Facebook would involve many unexpected issues.	
	PR3	Facebook may use my personal information inappropriately.	
Privacy value	PV1	To me, keeping my privacy online is the most important thing.	[45,46,48]
	PV2	I see greater importance in keeping personal information private compared to others.	
	PV3	I am more concerned with potential threats to my personal privacy compared to others.	
Trust in Facebook	TR1	Facebook is a trustworthy OSN.	[48,49,70]
	TR2	Facebook has a good reputation.	
	TR3	I can conduct my privacy on Facebook.	
	TR4	I will recommend that others use Facebook.	
Self-disclosure	SD1	My profile on Facebook is filled with details. *	[22,50,51,52]
	SD2	My profile on Facebook tells a lot about me. *	
	SD3	On Facebook, I disclose a lot of information about me. *	
	SD4	I am prepared to provide personal information on OSN. *	
	SD5	I am forced to submit personal information on OSNs.	
Privacy control	PCt1	I think I have control over who can access my personal information that is collected by Facebook.	[44,45,46]
	PCt2	I think I control who has access to my personal information on Facebook.	
	PCt3	I think I have control over how Facebook uses personal information.	
	PCt4	I think I can control my personal information which I provide to Facebook.	
Privacy concerns	PCs1	It upsets me when I have to put personal information on Facebook.	[24,45,46,48,49,71]
	PCs2	I am concerned that too much personal information about me is being collected by OSNs.	
	PCs3	I’m concerned that my personal information could be accessed by unauthorized people.	
	PCs4	I am concerned that there may be misuse of the information I submit on Facebook.	
	PCs5	I am concerned when I have to submit information on OSNs.	

* Reverse coded item.

**Table 4:** Factor loadings and Cronbach’s alpha.

Item	Factor Loading	Constructs	No. of Items	Cronbach’s α
PR1	0.824	Privacy risk	3	0.800
PR2	0.852			
PR3	0.603			
PV2	0.452	Privacy value	2	0.772
PV3	0.484			
TR1	0.843	Trust in Facebook	3	0.824
TR2	0.726			
TR3	0.770			
SD1	0.775	Self-disclosure	4	0.851
SD2	0.858			
SD3	0.789			
SD4	0.608			
PCt1	0.987	Privacy control	2	0.788
PCt2	0.683			
PCs1	0.756	Privacy concerns	5	0.901
PCs2	0.902			
PCs3	0.755			
PCs4	0.714			
PCs5	0.834			

**Table 5:** The measurement model fit test results fit.

Notation	Recommended Value	Measurement Model
GFI	≥0.90	0.903
CFI	≥0.90	0.923
NFI	≥0.90	0.903
RMSEA	≤0.10	0.074

**Table 6:** Composite Reliability (CR), Average Variance Extracted (AVE), and factor correlations matrix.

	CR	AVE	Privacy Risk	Privacy Value	Trust in Facebook	Self-Disclosure	Privacy Control	Privacy Concerns
**Privacy risk**	0.881	0.587	**0.766**					
**Privacy value**	0.864	0.642	0.314	**0.801**				
**Trust in Facebook**	0.893	0.615	0.130	0.003	**0.784**			
**Self-disclosure**	0.910	0.594	0.047	0.041	0.062	**0.771**		
**Privacy control**	0.883	0.689	0.020	0.003	0.228	0.001	**0.830**	
**Privacy concerns**	0.944	0.654	0.401	0.215	0.157	0.015	0.031	**0.809**

The bold diagonal elements represent AVE’s square root.

**Table 7:** The results of the testing of hypotheses.

H	Path			Standardized Coefficient β	T-Statistic	The Result
H1a	PR	-	TR	−0.413 ***	−6.851	Accepted
H1b	PR	-	PCs	0.627 ***	7.711	Accepted
H2	PV	-	PCs	0.231 ***	4.118	Accepted
H3a	TR	-	SD	−0.284 ***	−5.183	Accepted
H3b	TR	-	PCt	0.355 ***	7.528	Accepted
H3c	TR	-	PCs	−0.274 ***	−5.525	Accepted

*** *p* < 0.001.
